# Comparison of efficacy of rifaximin, probiotics and l-ornithine l-aspartate in overt hepatic encephalopathy: a randomized, phase IV, lactulose controlled clinical trial

**DOI:** 10.1186/s13063-025-09173-2

**Published:** 2025-11-21

**Authors:** Amna Shahbaz, QurratulAin Jamil, Shahid Muhammad Iqbal, Muhammad Nauman Jamil, Jawad Akbar Khan, Mohammed Aufy

**Affiliations:** 1 Department of Pharmacy Practice, Faculty of Pharmacy, The Islamia University of Bahawalpur, Bahawalpur, 63100 Pakistan; 2 Department of Pharmacology, Faculty of Pharmacy, The Islamia University of Bahawalpur, Bahawalpur, 63100 Pakistan; 3 The Children’s Hospital, The University of Child Health Sciences, Lahore, 54000 Pakistan; 4 Riphah Institute of Pharmaceutical Sciences, Riphah International University, Gulberg Campus, Lahore, 54660 Pakistan; 5 Division of Pharmacology and Toxicology, University of Vienna, Vienna, 1090 Austria

**Keywords:** Hepatic encephalopathy, West haven criteria, Liver cirrhosis, Treatment efficacy, Randomized control trial

## Abstract

**Background:**

Pakistan has a high prevalence of HCV and HBV, causing cirrhosis, leading to hepatic encephalopathy in approximately 30–45% of cirrhotic patients. Overt hepatic encephalopathy (OHE) is a serious type of hepatic encephalopathy that refers to brain dysfunction resulting from acute or chronic liver disease. Patients present flapping tremors and mental alterations leading to a coma, which disrupts daily activities and patient health-related quality of life. OHE can be treated by lactulose, rifaximin, probiotics, and l-ornithine l-aspartate (LOLA). Lactulose (controlled drug for trial) is the main treatment for OHE, even though its effectiveness in clinical trials has remained varied. Rifaximin (interventional drug), probiotics (interventional drug), and LOLA (interventional drug) all showed a significant effect in reducing the grade of hepatic encephalopathy, lowering blood ammonia levels, and enhancing psychomotor function in OHE patients. Our trial aims to determine the most effective treatment combination, as despite the availability of multiple treatment options, the efficacy is still uncertain. Limited studies have compared individual treatments, but no research has been conducted to assess all four treatment groups together, i.e., Group A (lactulose) as the control group, Group B (rifaximin + lactulose), Group C (probiotics + lactulose), and Group D (LOLA + lactulose).

**Methods:**

This trial is a single-center, parallel, multi-arm, randomized, unblinded, lactulose-controlled clinical trial. A total of 252 patients (both male and female aged 18–80 years), 63 in each treatment group, will be recruited in the study from the time of participation in the trial until the end of treatment (days 1–5). Primary outcome is to assess grade reversal of OHE on day 5 of trial participation. The secondary outcome is to determine the length of hospital stay and recovery time (days), monitoring of adverse drug reactions and death by cause in OHE patients.

**Discussion:**

This randomized controlled trial protocol will compare the efficacy of four proposed groups of medications to fill the gap in current knowledge.

**Trial registration:**

Chinese Clinical Trial Registry ChiCTR2300075925, registered on 19 September 2023; ClinicalTrials.gov NCT07037394, registered on 24 June 2025. Released to the public on 26 June 2025.

**Supplementary Information:**

The online version contains supplementary material available at 10.1186/s13063-025-09173-2.

## Administrative information

Note: The numbers in curly brackets in this protocol refer to the SPIRIT checklist item numbers. The order of the items has been modified to group similar items https://www.equatornetwork.org/reporting-guidelines/spirit-2013-statement-defining-standard-protocol-items-for-clinical-trials/.

**Table:** 

Title {1}	Comparison of Efficacy of Rifaximin, Probiotics and LOLA (L-Ornithine L-Aspartate) in Overt Hepatic Encephalopathy: A Randomized, Phase-IV, Lactulose Controlled Clinical Trial. (COPE-RPLL)
Trial registration {2a and 2b}	Chinese clinical trial registry ChiCTR2300075925, Registered on 19th of September 2023, https://www.chictr.org.cn/ ClinicalTrials.gov identifier: NCT07037394, Registered on 24 June 2025, https://clinicaltrials.gov/search?term=NCT07037394
Protocol version {3}	Our trial protocol version 2.0 is dated September 28, 2023.
Author details {5a}	COPE-RPLL trial collaborative group
Name and contact information for trial sponsor {5b}	Department of Pharmacy Practice, Faculty of Pharmacy, The Islamia University of Bahawalpur, Khawaja Fareed Campus, Pakistan, website/email: https://www.iub.edu.pk/
Role of sponsor {5c}	The sponsor and funder have no direct role in the design of study, the writing of protocol nor the decision to submit report for publication, the sponsor of study is Department of Pharmacy Practice, Faculty of Pharmacy, The Islamia University of Bahawalpur, represented by PI of the study who is Dr. QurratulAin Jamil responsible for every step of the trial.

## Introduction

### Background and rationale {6a}

Chronic liver disease (CLD) is defined as a degeneration in liver function which lasts for more than 6 months. Normal liver function includes the production of clotting factors and other proteins, bile excretion, and elimination of harmful metabolic products. Patients with CLD suffer from ongoing liver tissue inflammation, injury, and regeneration, and which results in the development of fibrosis and cirrhosis [1]. In Pakistan, CLD accounts for the fifth most common cause of death and the eleventh most common cause of disability [2]. The primary cause of CLD is the hepatitis B and C viruses [3] with a prevalence of 10 and 11.5% in adult population, respectively. Pakistan is ranked second in hepatitis C virus (HCV) infections, with almost one out of every 20 persons infected with HCV [4, 5] categorizing it as a nation suffering from cirrhosis [6].

During cirrhosis, liver is damaged, and with time, healthy liver tissue is replaced with a scar tissue, causing blood flow hinderance and decline in normal functioning [7]. It is the most frequent cause of hospital admission and death among the Pakistani population posing a significant strain on healthcare facilities reaching to epidemic levels [8]. Liver cirrhosis is categorized into compensated and decompensated stage. The median survival time for patients with compensated cirrhosis is over 12 years, and they do not present any symptoms. The main predictive factor for compensated cirrhosis is the presence of varices [9]. In contrast, individuals with decompensated cirrhosis have a median survival time of about 2 years and present at least one complication such as jaundice, ascites, variceal bleeding, or hepatic encephalopathy (HE). HE is one of the most serious and challenging complications of decompensated liver cirrhosis and defined as “brain dysfunction caused by liver failure or portal-systemic blood shunting that produces a spectrum of neurological/psychiatric abnormalities ranging from subclinical alterations to coma” [10]. This debilitating impairment linked with HE has a significant impact on the quality of life (QoL) for both patients and their caregivers [11, 12]. HE is classified as covert (minimal HE) and overt. Covert means clinically not visible, the patient appears stable with mild mental abnormalities, while overt means clinically visible, and patient exhibits a broad range of mental and motor dysfunction [13, 14]. Generally, the prevalence and incidence of HE is obscure due to variations in its symptoms, ranging from mild neuropsychological dysfunction to deep coma [10]. The occurrence of HE could be significantly elevated when transjugular intrahepatic portosystemic shunt (TIPS) is present [10]. Decompensation can get better to a compensated stage if the underlying cause of the liver disease is treated [9].

The approach for the management of HE is influenced by its severity which is evaluated by a five-point impairment scale referred to as the West Haven Criteria or Conn score. This scale grades HE from 0 (undetectable changes in personality or level of consciousness) to grade 4 (coma) [12]. Patients in grade 0–1 are included in minimal hepatic encephalopathy (MHE), while grade 2–4 are included in overt hepatic encephalopathy (OHE). Depending on severity, OHE can be treated by non-absorbable disaccharides (i.e., Lactulose), antibiotics (i.e., Rifaximin), and l-ornithine l-aspartate (LOLA) [15]. Lactulose (controlled drug) is a primary treatment for OHE despite its inconsistent effectiveness in clinical trials [16]. Rifaximin (interventional drug) have shown efficacy in treating OHE grade ≥ 1 [17–20]. Combining rifaximin with lactulose lowers the risk of HE related hospitalizations compared to lactulose alone, without diminishing its tolerability, with no severe side effects [21]. Probiotics (interventional drug) are cost-effective and have shown effectiveness in lowering blood ammonia levels, treating and preventing HE [22–24]. However, these trials have limitations, including limited number of participants, short treatment periods, uncertainty regarding the most efficacious strain from multiple strains of probiotics available in the market, their optimal dose, and variability in the results associated with HE [25, 26]. In clinical trials, both intravenous and oral administration of LOLA (interventional drug) demonstrated a statistically significant impact in reducing the HE grade, lowering blood ammonia levels and improving psychomotor function in cirrhotic patients and those suffering from mild or persistent form of HE compared to a placebo [27].

The availability of these four different treatment options for OHE motivated us to compare lactulose alone (Group A) with three combination therapies rifaximin + lactulose (Group B), probiotics + lactulose (Group C), and LOLA + lactulose (Group D) to find the most effective combination therapy among these available options.

### Objectives {7}

This clinical trial aims to compare the efficacy of four treatment groups, i.e., Group A (lactulose) as the control group, Group B (rifaxamin + lactulose), Group C (probiotics + lactulose), and Group D (LOLA + lactulose) for the treatment of OHE.

#### Primary objective

The primary objective will be to compare the grade reversal in OHE patients from the time of trial participation till the end of trial treatment (day 5) according to West Haven Criteria in both control and interventional groups.

#### Secondary objectives

i. To compare the length of hospital stay and recovery time (in days) from HE in both control and interventional groups.
ii. Adverse drug reactions monitoring in both control and interventional groups.
iii. To assess the mortality ratio in both control and interventional groups.

### Trial design {8}

**Fig. 1:**
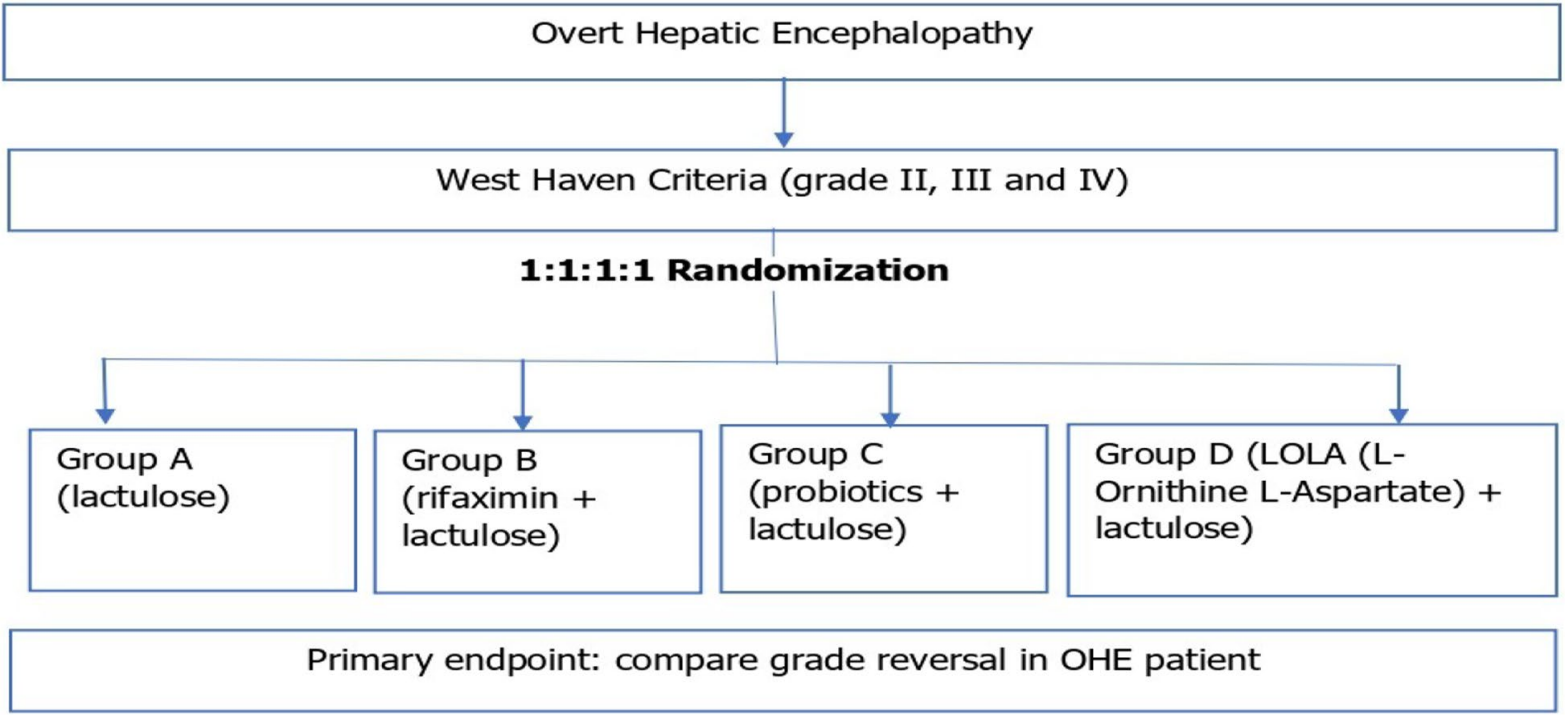
Graphical presentation of trial design

This trial is a single-center, parallel, multi-arm, 1:1:1:1 randomised, unblinded, lactulose-controlled superiority clinical trial comparing the efficacy of four groups, which are Group A (lactulose) as the control group, Group B (rifaximin + lactulose), Group C (probiotics + lactulose), and Group D (LOLA + lactulose) for the treatment of OHE (Fig. 1).

#### Protocol

The standard protocol items, which provide recommendations for guidelines about interventional trials, were outlined in this protocol. For the complete SPIRIT checklist, see https://www.equator.network.org/reporting-guidelines/spirit-2013-statement-defining-standard-protocol-items-for-clinical-trials/.

#### Framework/hypothesis

The experimental Group B (rifaximin + lactulose), Group C (probiotics + lactulose), and Group D (LOLA + lactulose) are superior to control Group A lactulose in the reversal of HE grades in patients of OHE.

## Methods: participants, intervention and outcomes

### Study setting {9}

This single-center trial will be conducted at Bahawal Victoria Hospital (BVH), a tertiary care hospital in Bahawalpur, Punjab, Pakistan. Our study will be conducted in several departments of BVH, including the Department of Gastroenterology and Hepatology unit, and other medical wards M1, M2, M3 and M4 [28].

### Eligibility criteria {10}

#### Inclusion criteria

1. Both male and female patients aged > 18 to < 80 years old at the time of consent.
2. Patients with decompensated chronic liver disease (DCLD) confirmed by ultrasound, endoscopy or blood tests associated with OHE grade II–IV according to West Haven Criteria will be included.
3. For patients who are unable to talk, their informed consent will be collected from their first-degree relative and/or legally authorized representative (LAR).
4. The patients/LARs have signed the consent/assent form authorized by the ethics committee, are willing to follow the treatment protocol, and have been informed about the purpose of the study.

#### Exclusion criteria

1. Patients aged ≤ 18 years old and ≥ 80 years old and those with grade 1 covert HE according to West Haven Criteria.
2. Patients with stroke, brain tumor, ventricular dysfunction, pulmonary edema, diarrhea, hepatocellular carcinoma, encephalitis (viral or bacterial), Wernicke and uremic encephalopathy, neurodegenerative disease (epilepsy, Parkinson’s disease), major psychiatric illness (schizophrenia), HIV-AIDS, drug intoxication, and recent use of sedatives or antidepressants.
3. The patient already participating in another drug trial.
4. Pregnant women will not be included.
5. Patients hypersensitive to lactulose, rifaximin, LOLA, and probiotics or any of its excipients will be excluded.

### Consent or assent {26a}

Following the Declaration of Helsinki and national regulations, a written informed consent will be obtained from all subjects who meet the inclusion criteria of our study [29]. For patients who are unable to talk or struggle to comprehend the study protocol and its procedures, their informed consent will be collected from first-degree relatives and/or LAR. During the consent or assent process, the investigators or their representatives must provide information about all important aspects of the study, including any pros and cons of enrollment in the trial to the patient or their first-degree relative or LAR, respectively. Any patient who withdraws prior informed consent, their data collected until the withdrawal point from the consent, will be included for analysis.

### Additional consent provisions for collection and use of participant data and biological specimens {26b}

N/A-in our trial, there will be no collection of biological samples. Consent will be obtained for the clinical trial team to assess patient medical records and publish the data in aggregate form without revealing the identity of the patient.

### Interventions

#### Explanation for the choice of comparators {6b}

Selection of our trial groups (control and intervention) will be done by choosing the same inclusion and exclusion criteria, and both groups will follow the usual care for treating OHE with lactulose. The interventional groups will also be treated with lactulose in combination, i.e., (rifaximin + lactulose), (probiotics + lactulose), and (LOLA + lactulose). There are several treatment options for HE, but lactulose is still recommended as the primary treatment. The doses of the trial drugs were kept the same according to guidelines with established safety and tolerability [8, 10, 30].

### Intervention description {11a}

#### Name and description of trial drugs

1. Lactulose
  Lactulose is a synthetic, non-absorbable sugar, a liquid laxative and is the primary treatment option for both MHE and OHE [31]. Its mechanism of action includes the breakdown of lactulose in the colon by gut bacteria into acetic and lactic acid. This changes gut pH to acidic, thus promoting excretion of NH_3_ by conversion into non-absorbable NH_4_^+^, which lowers ammonia levels in the blood [32].
2. Rifaximin
  Rifaximin is a non-absorbable oral antibiotic that is selective to the gut. It is effective against a broad spectrum of Gram-positive and Gram-negative organisms, as well as anaerobic enteric bacteria [12, 33]. Rifaximin is approved by the U.S. Food and Drug Administration (FDA) solely for the secondary prevention of OHE. However, in a scenario where there is suspicion of MHE, even without confirmation, the treatment may involve starting non-absorbable disaccharides (and/or rifaximin) [14]. Rifaximin exhibits efficacy similar to lactulose and is used in combination (rifaximin + lactulose) for treating OHE [31].
3. Probiotics
  Probiotics contribute to the treatment of HE by modifying the gut microbiota, reducing the presence of harmful bacteria, acidifying the intestinal mucosa, reducing the production and absorption of ammonia, and lowering endotoxin levels. Therefore, enhancing intestinal barrier function and improving the nutritional health of the gut epithelium [25, 26]. Several studies have demonstrated the effectiveness of probiotics in lowering blood ammonia levels, treating MHE and preventing OHE [25].
4. LOLA
  LOLA can reduce levels of ammonia in the blood by promoting the urea cycle and glutamine synthesis in periportal hepatocytes. Intravenous LOLA can be administered as an add-on therapy for patients who are unresponsive to standard treatment, but additional research is needed to establish its appropriate dosage and duration [10]. It is reported that administration of intravenous LOLA has more favorable outcomes compared to oral LOLA across the spectrum of HE [16].

### Drug administration and dosage schedule

After randomisation, treatment will be initiated in OHE patients (252), included in our trial. In each treatment group, 63 patients will be included who will receive either Group A (lactulose) as control, Group B (rifaximin + lactulose), Group C (probiotics + lactulose), or Group D (LOLA + lactulose).

- Group A: lactulose syrup (120 ml).
  Dose: 30 ml three times daily, orally or by nasogastric tube (NG) for 5 days [8]
- Group B: Rifaximin tablet (550 mg) + lactulose syrup.
  Dose: 1 tablet twice daily, orally or by nasogastric tube (NG) with 30 ml of lactulose syrup three times daily, orally or by nasogastric tube (NG) for 5 days [8]
- Group C: Probiotic sachet (2 g) + lactulose syrup.
  Dose: 1 sachet twice daily, orally or by nasogastric tube (NG) with 30 ml of lactulose syrup three times daily, orally or by nasogastric tube (NG) for 5 days [34, 35]
- Group D: LOLA concentrate for infusion (0.5 g/ml) + lactulose.
  Dose: 4 ampoules (40 ml) of LOLA (20 g) diluted in 460 ml of 5% dextrose administered as an IV infusion at a rate of 21 ml/h over 24 h for 5 days with 30 ml of lactulose syrup three times daily, orally or by nasogastric tube (NG) for 5 days [36].

### Drug storage and supply

Drugs will be stocked securely in separate cabinets of satellite pharmacies of the gastroenterology unit and in medical wards (M1, M2, M3, and M4) where they will always accessible to the trial pharmacist for carrying out the trial. All trial medications will be stored in a cool and dry place.

### Relevant concomitant care permitted or prohibited during trial {11d}

Standard of care (SOC) treatment will be given to all participants of our study groups (control and intervention). There are no limitations on the use of additional medications. Trial participation will not lead to any necessary treatment being prohibited.

### Provision for post-trial care {30}

There are no anticipated risks associated with our trial interventional drugs, as these are used in routine clinical practice. Thus, no special arrangements are provided for additional care during or after the trial. During the trial, we will consistently track the survival rates, complications and the safety of the treatment across all study groups to identify any harm associated with lactulose or other treatment groups.

### Strategies to improve adherence to interventions {11c}

The trial pharmacist will monitor adherence to the protocol, i.e., by recording time and date of trial treatment administration till the end of the trial (day 5). Furthermore, a treatment log will be maintained by noting down the number of trial medications provided vs. the number of trial medications returned (empty packs of medicines or unused). Moreover, SOC treatment for every patient will be maintained and will be independent of our trial interventions.

### Criteria for discontinuing or modifying allocated interventions {11b}

In case of disease condition worsening, patients will be shifted to the best possible treatment for curing OHE according to the updated guidelines. In case of any harm or contraindication during the trial treatment, intervention will be stopped and discontinued. Patient data will be gathered until he/she remains in the study and is recorded in the case report form (CRF).

## Outcomes {12}

### Primary outcome

To compare, the efficacy of Group B (rifaximin + lactulose), Group C (probiotics + lactulose), and Group D (LOLA + lactulose) with Group A (lactulose) for the treatment of OHE. Reversal to lower grade is examined from the time of participation in the trial until the end of treatment (day 5) according to West Haven Criteria.

### Secondary outcomes

1. To determine the length of in-hospital stay (days)
2. Time of recovery from encephalopathy (days)/(time taken in days to recover from grade 2 or above according to WHC to grade 1 or below)
3. The severity of liver disease will be evaluated by the Child–Pugh class.

## Sample size {14}

### Size of the treatment effect

Based on existing studies, it is estimated that there is a 79% cure rate using LOLA, a 76.2% cure rate using rifaximin, a 65.6% cure rate using probiotics, and a 55.6% cure rate using lactulose for treating HE patients [30, 36–38]. So, by the given formula [39], we estimated our sample size using all the cure rates of our study groups (control and intervention).

The formula used for sample size calculation is:

$$n=\left[{\left({Z}_{\alpha/2}+{Z}_{\beta }\right)}^{2}\times \left[p1\left(1-p1\right)\right]+p2\left(1-p2\right)/{\left(p1-p2\right)}^{2}\right]$$

where,

*n* = sample size required in each group,

p1 = proportion of subjects cured by LOLA = 0.79 [36]

p2 = proportion of subjects cured by Lactulose = 0.556 [36]

p1-p2 = clinically significant difference = 0.23.

Z _α/2_: This depends on the level of significance; for 5% this is 1.96.

Z_β_: This depends on power, for 80% this is 0.84.

Based on a given formula, the sample size required per group is 60, with a total sample size of 240 patients. Assuming the dropout rate of 5%, we will enroll 252 patients, i.e., 63 patients in each treatment group.

The description of the sample size in the protocol will be:

- A sample size of 252 patients, 63 in each treatment group, will be sufficient to detect a clinically important difference of 23% between groups in curing HE by using a two-tailed z-test of proportions between two groups with 80% power and a 5% level of significance. This 23% difference represents a 79% cure rate using LOLA and a 55.6% cure rate using lactulose.

### Recruitment {15}

All patients who have given consent to participate in this trial and are diagnosed by attending physicians with OHE will be recruited into our study groups (control and intervention).

### Participant timeline {13}

**Fig. 2:**
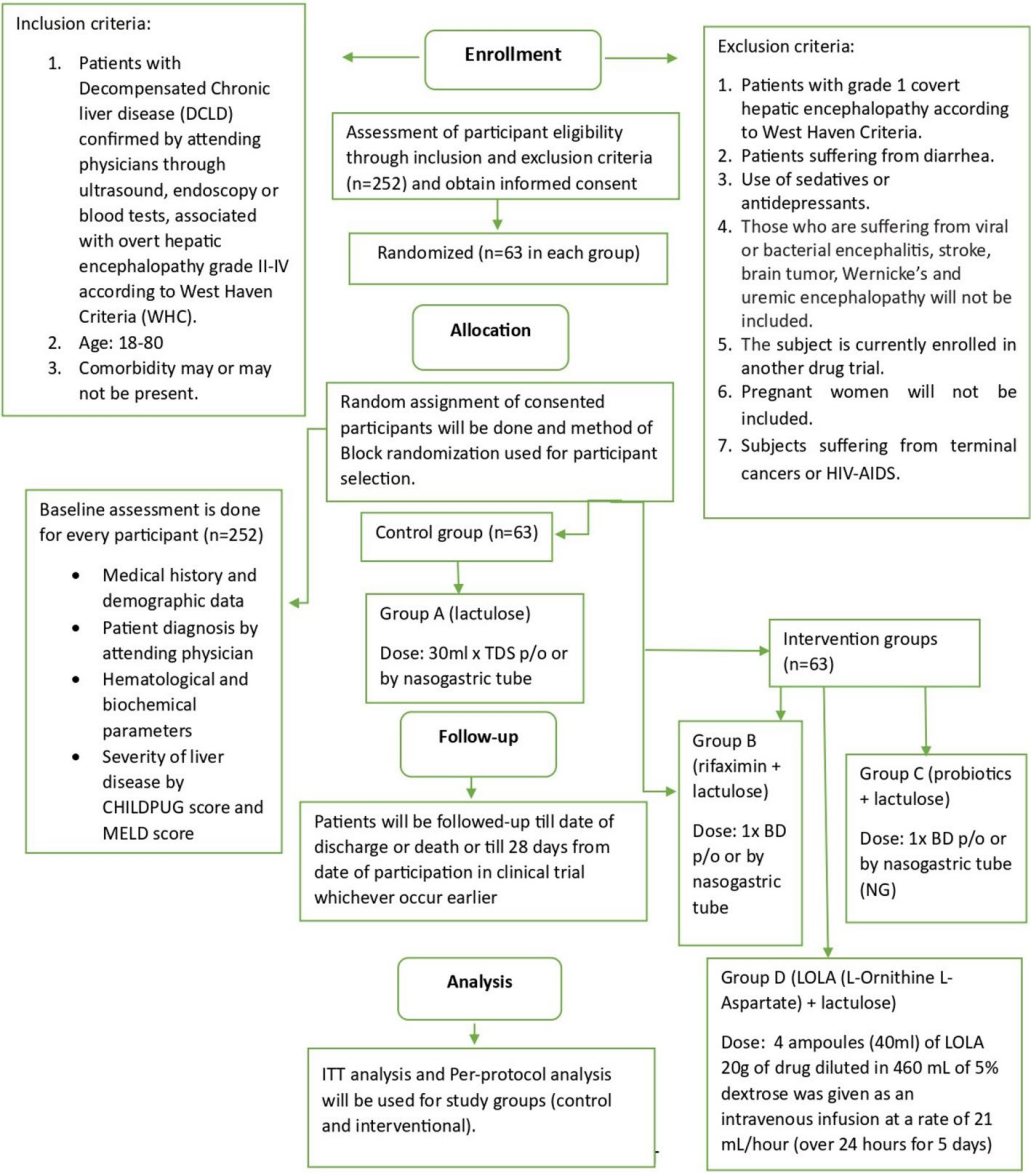
Participant timeline of trial events

**Fig. 3:**
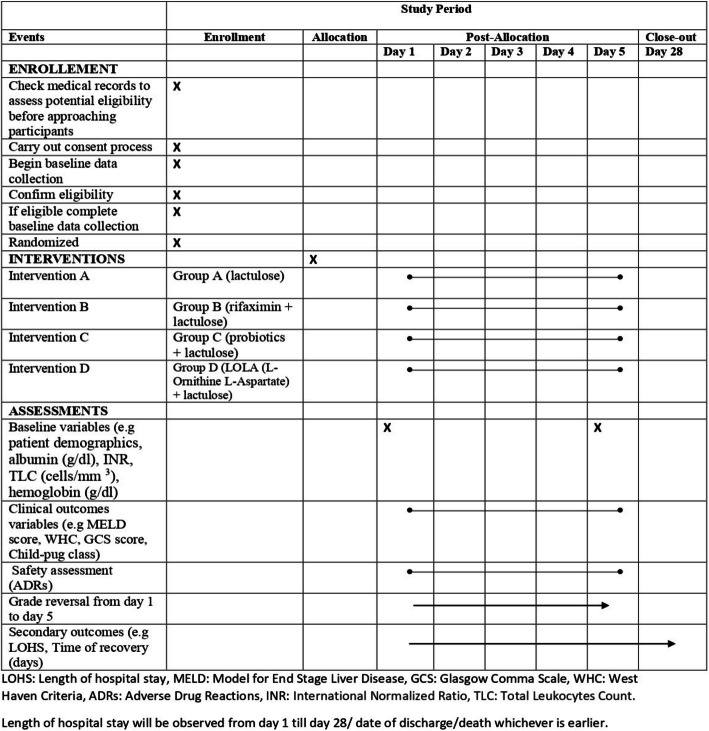
Schedule of trial events

The timeline of participants is presented in Fig. 2, and the schedule of events in Fig. 3.

## Assignment of interventions: allocation

### Allocation: sequence generation {16a}

HE patients will be randomized in a 1:1:1:1 allocation to Group A (lactulose) as the control group, Group B (rifaximin + lactulose), Group C (probiotics + lactulose), and Group D (LOLA + lactulose). Block randomization is carried out using permuted blocks of size 21, strata of 3 (Grade-2, Grade-3, and Grade-4 of West Haven Criteria) by using a sealed envelope (https://www.sealedenvelope.com/simple-randomiser/v1/lists) [36]. Block randomization is used to ensure that the allocation ratio is maintained throughout the trial.

### Allocation: concealment mechanism {16b}

Concealment of randomization sequence will be carried out by using an online tool which creates a randomization list for trial participants (see “Allocation: sequence generation” {16a} section). An opaque, sealed envelope will be used for assigning treatment to patients. This list will not be accessible to the trial patients and physicians until the recruitment of patients in the trial.

### Allocation: implementation {16c}

A statistician, independent of the enrollment and analysis team, will be responsible for sequence generation. Once a patient is confirmed with OHE and is fulfilling the inclusion criteria. Then, a member of the enrollment team will be informed, and that member will assess patient inclusion and exclusion criteria and informed consent/assent will be obtained from the patient or from the patient's first-degree relative/LAR.

**Fig. 4:**
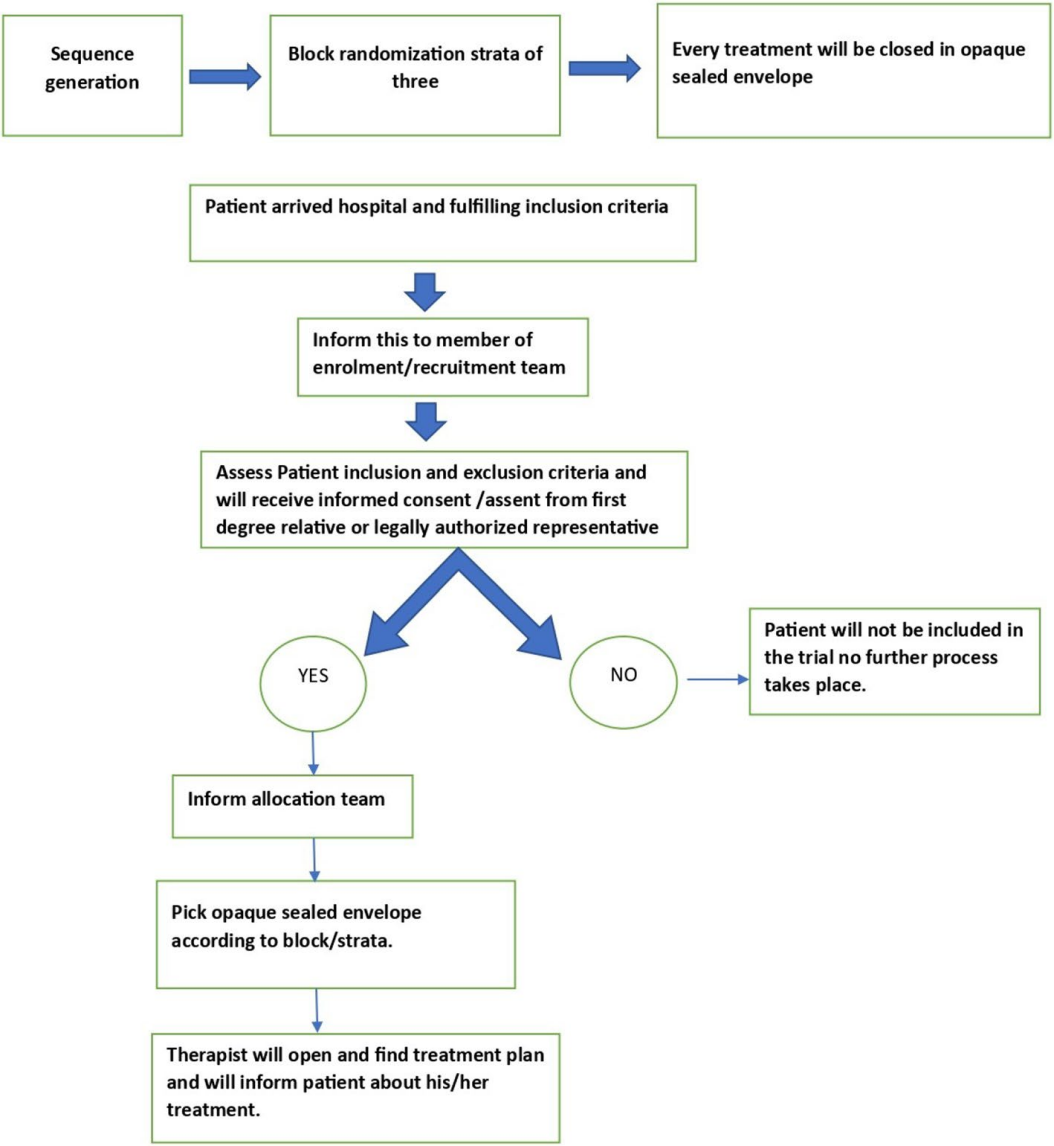
Allocation sequence generation and implementation

According to relative strata (Grade-2, Grade-3, and Grade-4 of West Haven Criteria), a trial pharmacist from the allocation team will pick up the opaque sealed envelope and will give it to the therapist. The therapist will open the envelope and will inform treatment plan to the patient. The trial pharmacist will provide trial medicines to the qualified person. A treatment pack will be administered to that patient by a qualified person (nurses, physician) under the supervision of a trial pharmacist according to the treatment plan enclosed in an envelope. The randomization list will be stored securely by an independent statistician responsible for sequence generation. Only the trial pharmacist will have access to the opaque sealed envelope for treatment assignment (Fig. 4).

## Assignment of interventions: blinding

### Who will be blinded {17a}

The trial is open-label. Blinding of the attending physicians, trial pharmacist, or study subjects is not feasible due to the intervention design.

#### Procedure for unblinding if needed {17b}

Not applicable as the trial is open-label and does not require any blinding.

## Data collection and management

### Plans for assessment and collection of outcomes {18a}

#### Baseline

After the completion of the patient consent procedure, the baseline data of the participant will be collected and entered by the trial team onto a paper CRF and subsequently into an electronic database. The trial staff authorized for the collection of the trial sample and data of trial will be trained in the sample and data collection procedure.

Baseline data includes patient demographics, i.e., age, income, level of education, socio-economic class, and any other risk factor or comorbidity associated with OHE. Additionally, laboratory parameters will be collected, which include, e.g., albumin (g/dl), international normalized ratio (INR), TLC (cells/mm^3^), hemoglobin (g/dl), platelet count (cells/mm^3^), liver function tests (LFTs), RFTs, and serum electrolytes: sodium (mmol/l), potassium (mmol/l) levels. Furthermore, to assess the severity of liver disease, we will use the Child–Pugh class.

## Assessments

### Primary outcome

Efficacy of drugs will be assessed by a validated questionnaire (case report form) which uses the pre-validated tool, West Haven Criteria for assessing grade reversal.

### Secondary outcomes

Liver disease severity will be assessed by the Child–Pugh class. We will find the length of in-hospital stay (days) and time of recovery from OHE (days).

#### Plans to promote participant retention and complete follow-up {18b}

Patient follow-up will occur in the hospital, and there is no requirement for strategies to enhance retention due to the minimal follow-up period. During the trial intervention, patient will be followed up for 5 days, and after that patient will be followed up till the date of discharge or death or till 28 days from the date of participation in the clinical trial, whichever occurs earlier.

#### Withdrawal

A patient can withdraw from the trial whenever he/she wants, and he/she may or may not justify the cause of withdrawal. If a patient leaves the trial, we will examine the data obtained until the patient leaves, but we will not collect any more data unless the patient consents. In any situation, we will honor the patient’s preferences. In case of non-adherence, we will collect patient data, and he/she will remain in the trial and will not be “off-study.”

### Data management {19}

**Fig. 5:**
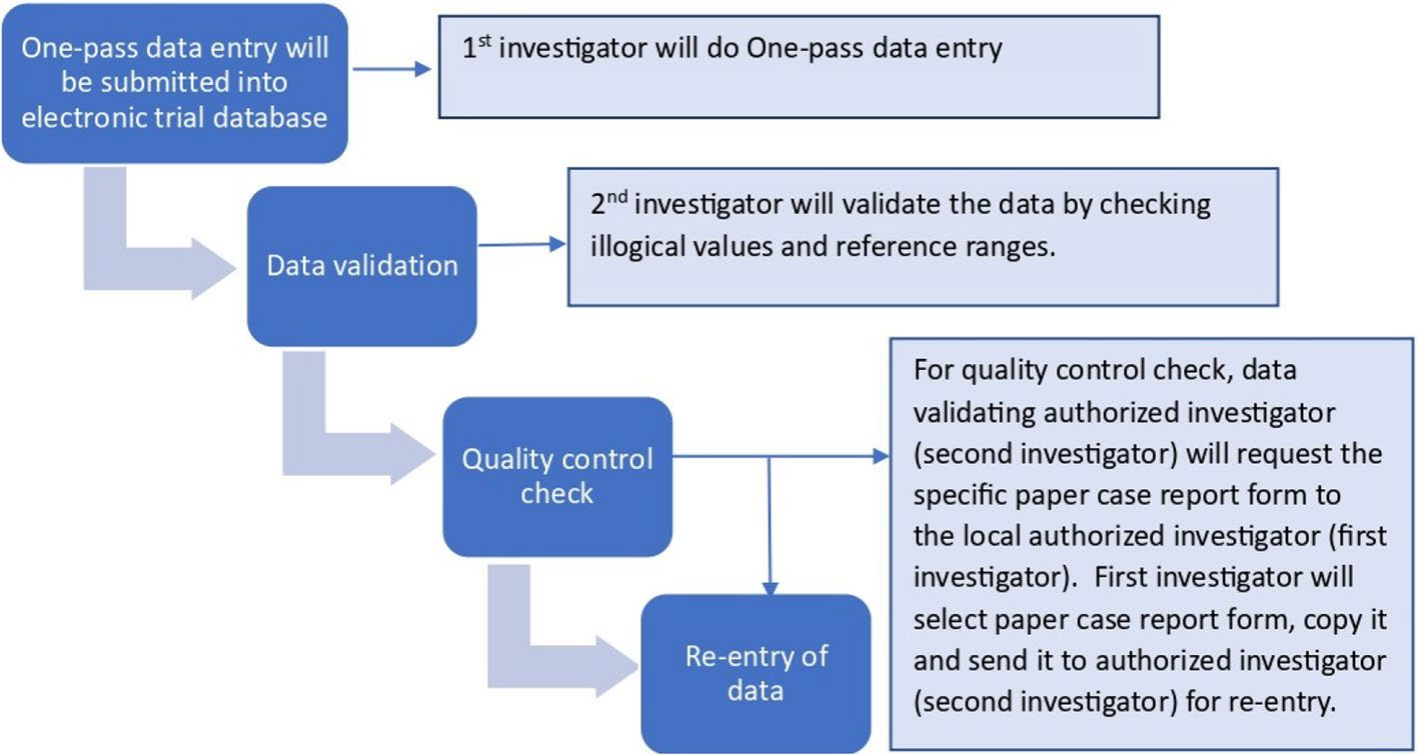
Data management process of the trial

One-pass data entry will be submitted into the electronic trial database upon completion of the paper case report format at our study site by a local authorized investigator (first investigator). A unique ID and password will be used to access the trial database (Fig. 5).

Another authorized investigator (second investigator) will validate the data by checking illogical values and reference ranges. The data which is missing will be collected and displayed as original without any imputation. For raw and non-numeric data, codes with their description will be provided where required.

Any discrepancies or errors addressed in the trial database will be inquired by the second investigator by requesting a scanned copy of the paper case report form, and query will not be closed until resolved. Data modification will be documented after each query. Before entering trial data into the database, checks will be applied for correct data entry.

For quality control checks and data validation, the authorized investigator (second investigator) will request the specific paper case report form from the local authorized investigator (first investigator). The first investigator will select a paper case report form, copy it, and send it to the authorized investigator (second investigator) for re-entry. Entry, processing, and evaluation of data will be compiled according to International Council for Harmonisation of Technical Requirements for Pharmaceuticals for Human Use (ICH) Guideline for good clinical practice E6 (R3).

#### Coding

Manual coding will be done. We will use the medical dictionary for regulatory activities (MedDRA) for coding adverse events and the cause of death. As a part of adverse drug reaction (ADR) reporting, concomitant medication data will be collected by utilizing the British National Formulary (BNF).

#### Data storage

Unique ID numbers of participants will be used for pseudonymized trial data. After that, data will be stored securely in the trial database. Electronic data used for data analysis and publication will not include any patient identification information.

Original paper case report forms, consent forms, and essential trial documents will be recorded and maintained in the investigator site file (ISF) present at the participating location securely. The principal investigator (PI) will keep the file updated throughout the trial. The file will be kept in a secure cabinet and will be accessible only to authorized investigators of the trial team. Arrangement of trial participant files will be done in sequential order and stored in a safe and easily accessible manner. Data and records of our clinical trial will be retained for a minimum of three years after the end of the trial.

Hospital records, including patient laboratory reports, medicine chart sheet, daily progress notes, medical history, past and current medication, patient clinical outcomes, and adverse events will be included in trial source documents.

#### Confidentiality {12}

Investigators, the allocation team, and all the personnel involved in trial management will keep every patient’s information confidential. Excluding the consent form, all trial documents and electronic databases, patients will be identified by their screening ID and randomization number. On paper, CRF patient initials will be added, and it will be kept at the participating site. All documents will be stored securely and will be accessible by authorized personnel only. Data protection will be conducted in compliance with the ICH guideline for good clinical practice E6(R3). The database of our trial will be accessed by a complex password ageing mechanism.

#### Plans for collection, laboratory evaluation, storage of biological specimens for genetic or molecular analysis in this trial/further use {33}

N/A—No biological specimens will be collected.

## Statistical methods

### Statistical methods for primary and secondary outcomes {20a}

#### Populations for analysis

All analyses will be conducted using the intention-to-treat (ITT) population, unless specified otherwise. Intention-to-treat patients will be evaluated based on the trial groups they were randomly assigned to, irrespective of the treatment they received, their eligibility for the study or their adherence after randomization. Any missing data will be assigned grade 4 of OHE when a patient is presented in a high mental state or grade 2 of OHE when the patient is presented in an average mental state, and calculations will be carried out accordingly.

The per-protocol population consists of all randomized patients who met the eligibility criteria for the study and who followed the assigned treatment for a minimum of 5 days after being randomized (adhered to our trial protocol fully). Patients who did not receive their assigned treatment or who withdrew from the trial will not be included.

#### Primary analysis

Primary analysis (intention-to-treat) will be applied to the primary outcome that is reversal in OHE grades, which is measured by West Haven Criteria from day 1 until day 5 of treatment allocation between all 4 treatment groups. The primary endpoint will also be analyzed in a primary analysis based on the per-protocol population.

#### Methods of analysis

A sensitivity analysis will be conducted using a complete case analysis (listwise deletion (LD) within the ITT population. Patients with missing data will not be included in the analysis, which supports the assumption that the data is missing at random.

### Primary outcome

#### Reversal of grade

The baseline mental state grade (reversal of grade from day 1–5) in ITT and per-protocol population will be analyzed by using an ordinal regression model. We will use the trial arm as an explanatory factor (with lactulose as the reference group).

### Secondary outcomes

#### Rate of recovery

The rate of recovery from OHE will be evaluated based on the number of days it takes to improve from a mental state grade of 2 or higher (3, 4) to a grade of 1 or lower (zero). Time of recovery between the trial groups is compared by using Cox regression analysis.

#### Length of hospital stay

The comparison between the length of hospital stay until discharge between trial arms will be analyzed by using a paired t-test and linear regression analysis.

#### Mortality

Mortality or no recovery from HE within 5 days will be examined using logistic regression. To ensure safety, we will closely monitor mortality during the trial. The criteria for early study termination are explained in the section titled “Early Termination of the Study.”

#### Demographics and baseline characteristics

Descriptive statistics will be applied to demographic and baseline characteristics, and all data will be summarized in tables and figures for all randomized groups. Continuous variables will be represented as frequency (*n*), standard deviation (SD), mean and median. In certain situations, missing data may be categorized separately based on the characteristics of the variable. Categorical variables will be presented by number and percentage in each category. All test analyses will be reported with 95% confidence intervals (CIs), and *p* values ≤ 0.05 will be taken as significant. All statistical tests and CIs will be two-tailed. The statistical analysis will be performed using SPSS (Statistics for Windows, version 21.0; IBM Corp., Armonk, NY).

### Interim analyses {21b}

No interim analysis will be applied to trial data, as our trial treatment is already approved by the regulatory agencies and recommended by several international guidelines for treatment of OHE, demonstrating minimum risks. Thus, the final analysis will take place once all trial data have been collected.

#### Methods for additional analyses (e.g., subgroup analysis) {20b}

Subgroup analyses will be scheduled to be performed on the primary outcome to assess differences between our trial arms. Subgroup variables include sociodemographic variables, comorbidities, and disease characteristics (presenting complaints, OHE grades, duration of disease, Child–Pugh class).

Randomization presents balanced treatment groups. However, the strata of subgroups may not be evenly distributed. Thus, during the randomization process, stratification of HE grades was included to ensure equal distribution among trial groups.

Certain baseline variables may be linked to subgroup variables and the effect of treatment. Potential confounders will be adjusted when necessary, and we will examine the relationship between baseline characteristics and OHE grades. Relative risk and CIs will be reported along with the *p* value from tests for interaction. Relative risk (RR) will be considered as the most reliable estimate for all subgroups until there is strong interactive evidence (*p* < 0.001).

### Methods in analysis to handle protocol non-adherence and any statistical method to handle missing data {20c}

We will conduct intention-to-treat and per-protocol analysis. Sensitivity analysis will be conducted by excluding participants who did not receive trial treatments (non-adherent patients). We will sum up any missing data for baseline and outcome variables, but will not use any technique to impute it because we expect that the amount of missing data will be insignificant.

### Plans to give access to full protocol, participant-level data and statistical code {31c}

Public access may be granted to the complete protocol, anonymized datasets of individual participants (upon valid request), and the statistical code (upon request) following the manuscript publication that details the trial results, following the journal’s guidelines.

## Methods: monitoring

### Data monitoring {21a}

The study will be conducted at a single-center, and the study team, led by the principal investigator, will oversee data monitoring. Trial interventional drugs are already approved in the guidelines and widely used in routine practice, so these have minimal risk. Due to minimal risk and a single-center study, there is no need for a data monitoring committee in this trial. The study team continuously oversees the study procedures, and the data collected. If there are indications that patients in the intervention group may be at risk from the study procedures, the study can be halted at any moment.

### Harms {22}

Harms will be monitored throughout the trial, our trial medicines are tested and approved in the guidelines, so the risk of hazards is very less. Early termination of the study occurs in case of any safety concerns, outlined in the section of “early termination of study.”

#### Early termination of study

The following conditions will be used for early study termination.

- There is a risk of developing adverse drug reactions in the patient
- Occurrence of an unknown adverse drug reaction
- Patient safety is doubtful or at risk

### Auditing {23}

In relation to monitoring the activities described above, there is no need for specific auditing for this single-center trial.

### Dissemination plans {31a}

The results of our trial will be published in peer-reviewed journals. Upon completion, our study results will be disseminated among the gastroenterology and hepatology specialists by including these results in national and international clinical practice guidelines.

## Ethics and dissemination

### Research ethics approval {24}

This study protocol is approved by the Pharmacy Human Ethics Committee (PHEC) of the Islamia University of Bahawalpur (187–2023-/PHEC).

### Protocol amendments {25}

All pertinent modifications to the protocol will be submitted to the Ethics Committee at the Islamia University of Bahawalpur for approval. No changes will be made until ethical approval is granted.

### Declaration of interest {28}

No declaration of interest is available for this trial.

### Data access {29}

All researchers and members of the trial team who participated in collecting and evaluating data, as well as publication writing, will have full access to all data collected. There are no binding agreements with any parties that would hinder access to the data in any manner.

## Discussion

The treatment of OHE involves lifestyle changes, i.e., protein restrictions on diet. Debilitating symptoms of OHE impair patient self-care, which results in poor diet consumption and non-compliance with treatment plans. Thus, leading to repeated hospitalizations and diminished QoL. There are several guidelines, including the American Association for the Study of Liver Diseases (AASLD), Italian Association for the Study of the Liver (AISF) and the Pakistan Society of Hepatology Guidelines on the Management of HE that recommend treatment for OHE, including lactulose, rifaximin, probiotics, and LOLA, respectively [8, 10, 27]. According to guidelines, the utilization of lactulose is considered as a mainstay of therapy for the treatment of OHE. However, it exhibits inconsistent effectiveness in various clinical trials [16]. Several guidelines suggest that rifaximin (an interventional drug) plays a positive role in the management of OHE [17–20]. According to the latest guidelines [22–24], probiotics (an interventional drug) have similar efficacy to lactulose in improving mental state in OHE patients. However, the data on probiotics in OHE is insufficient due to the limited number of participants, the variety of probiotics utilized and their inconsistent duration of treatment [26] so we aimed to assess the efficacy of probiotics as an adjunct to lactulose in treating OHE. Several studies demonstrate that intravenous LOLA (interventional drug) is significantly effective in the reversal of OHE grades [27].

The study is a single-center, confirming all standards of care for OHE treatment in all four trial arms. To ensure even distribution of each grade (II, III, IV) among trial groups, stratification of HE grades was included during randomization process.

The COPE-RPLL trial has a simple aim: to compare the efficacy of our trial medicines to find the most efficacious treatment combination in comparison to the standard of care for treating OHE. Up to 70% of individuals with cirrhosis experience HE, which is responsible for causing death in 30% of individuals suffering from CLD. This trial would serve as the first RCT to add further information to the available data.

### Trial status

This protocol was reported following the SPIRIT checklist [40]. Our trial protocol version 2.0 is dated September 28, 2023. Recruitment for the trial was started in December 2023 and will be completed by April 2025.

## Supplementary Information

Additional file 1. Consent Procedure Overview.
Additional file 2. Brief Information Sheet.
Additional file 3. Study Information for Participants.
Additional file 4. Informed Consent Form.

## Data Availability

All researchers and members of the trial team who participated in collecting and evaluating data, as well as publication writing, will have full access to all data collected. There are no binding agreements with any parties that would hinder access to the data in any manner.

## Abbreviations

AASLD: American Association for the Study of Liver Diseases
ADR: Adverse drug reaction
AISF: Italian Association for the Study of the Liver
BD: Twice a day
BNF: British National Formulary
BVH: Bahawal Victoria Hospital
CLD: Chronic liver disease
CRF: Case report form
DCLD: Decompansated Chronic Liver Disease
EASL: European Association for the Study of the Liver
FDA: U.S. Food and Drug Administration
GCP: Good clinical practice
HBV: Hepatitis B virus
HCC: Hepatocellular carcinoma
HCV: Hepatitis C virus
HE: Hepatic encephalopathy
ICH: International Council for Harmonisation of Technical Requirements for Pharmaceuticals for Human Use
INR: International normalized ratio
ISF: Investigator site file
LAR: Legally authorized representative
LFTs: Liver function tests
LOLA: L-ornithine L-aspartate
M1: Medical Ward 1
M2: Medical Ward 2
M3: Medical Ward 3
M4: Medical Ward 4
MedDRA: Medical Dictionary for Regulatory Activities
MELD: Model for End-Stage Liver Disease
MHE: Minimal hepatic encephlopathy
NG: Nasogastric tube
OHE: Overt hepatic encephalopathy
PHEC: Pharmacy Human Ethics Committee
QoL: Quality of life
RCT: Randomized controlled trial
RFTs: Renal function tests
SOC: Standard of care
SPIRIT: Standard Protocol Items: Recommendations for Interventional Trials
TDS: Three times a day
TIPS: Transjugular intrahepatic portosystemic shunt
TLC: Total leukocytes count
WHC: West Haven Criteria

## References

[1] Sharma A, Nagalli S. Chronic liver disease. In: StatPearls. Treasure Island (FL) ineligible companies: StatPearls Publishing LLC.; 2024. 32119484

[2] Majid B, Khan R, Junaid Z, Khurshid O, Rehman SH, Jaffri SN, Zaidi B, Zehra J, Batool S. Assessment of knowledge about the risk Factors of chronic liver disease in patients admitted in Civil Hospital Karachi. Cureus. 2019;11:e5945. 10.7759/cureus.5945 PMC6860746 31799087

[3] Qazi F, Khan SB. Hepatic encephalopathy in chronic liver disease: predisposing factors in a developing country. Asian J Med Sci. 2015;6:35.

[4] Anwar F, Khan M, Salman M, Ahmad S. Abdullah, Ullah F, Khan J, Haq I, Abbas M: Seroprevalence of hepatitis B virus in human population of district Buner Khyber Pakhtunkhwa Pakistan. Clin Epidemiol Glob Health. 2021;10:100688.

[5] Farhan M, Fazal F, Dave T, Butt A, Basit J, Maqbool S. National hepatitis registry in Pakistan: a dire need for hepatitis surveillance and control. Trop Med Health. 2023;51:41. 37542354 10.1186/s41182-023-00534-8 PMC10401767

[6] Qureshi SA, Shahbaz J, Abbasi S, Nazir R, Ahmed A. A two year experience of upper gastrointestinal endoscopy at Bahawal Victoria Hospital, Bahawalpur. Med Forum Mon. 2019;30:7–10.

[7] Definition & Facts for Cirrhosis. https://www.niddk.nih.gov/health-information/liver-disease/cirrhosis/definition-facts.

[8] Naeem MU, Malik K, Fareed A, Kashif R, Haider A, Ghilzai D, Ramzan HS. Pakistan society of hepatology guidelines on the management of hepatic encephalopathy: guidelines for managing hepatic encephalopathy. Pakistan J Health Sci. 2024;5:02–08.

[9] Viral hepatitis and liver disease. https://www.hepatitis.va.gov/cirrhosis/background/stages.asp.

[10] Montagnese S, Russo FP, Amodio P, Burra P, Gasbarrini A, Loguercio C, et al. Hepatic encephalopathy 2018: a clinical practice guideline by the Italian Association for the Study of the Liver (AISF). Dig Liver Dis. 2019;51:190–205. 30606696 10.1016/j.dld.2018.11.035

[11] Duah A, Agyei-Nkansah A, Osei-Poku F, Duah F, Ampofo-Boobi D. The prevalence, predictors, and in-hospital mortality of hepatic encephalopathy in patients with liver cirrhosis admitted at St. Dominic Hospital in Akwatia, Ghana. Can J Gastroenterol Hepatol. 2020;2020:8816522. 10.1155/2020/8816522 PMC7772042 33425806

[12] Iadevaia MD, Prete AD, Cesaro C, Gaeta L, Zulli C, Loguercio C. Rifaximin in the treatment of hepatic encephalopathy. Hepatic Med: Evid Res. 2011;28:109–117. 10.2147/HMER.S11988 PMC3846583 24367227

[13] Bajaj J, Cordoba J, Mullen K, Amodio P, Shawcross D, Butterworth R, et al. The design of clinical trials in hepatic encephalopathy—an International Society for Hepatic Encephalopathy and Nitrogen Metabolism (ISHEN) consensus statement. Aliment Pharmacol Ther. 2011;33:739–47. 21306407 10.1111/j.1365-2036.2011.04590.x PMC3971432

[14] Montagnese S, Rautou P-E, Romero-Gómez M, Larsen FS, Shawcross DL, Thabut D, et al. EASL clinical practice guidelines on the management of hepatic encephalopathy. J Hepatol. 2022;77:807–24. 35724930 10.1016/j.jhep.2022.06.001

[15] Strauss E, Tramote R, Silva E, Caly W, Honain N, Maffei R. Double-blind randomized clinical trial comparing neomycin and placebo in the treatment of exogenous hepatic encephalopathy. Hepatogastroenterology. 1992;39:542–5. 1483668

[16] Patidar KR, Bajaj JS. Covert and overt hepatic encephalopathy: diagnosis and management. Clin Gastroenterol Hepatol. 2015;13:2048–61. 26164219 10.1016/j.cgh.2015.06.039 PMC4618040

[17] Festi D, Mazzella G, Orsini M, Sottili S, Sangermano A, Bassi SL, et al. Rifaximin in the treatment of chronic hepatic encephalopathy; results of a multicenter study of efficacy and safety. Curr Ther Res. 1993;54:598–609.

[18] Massa P, Vallerino E. Dodero MJEJCR: Treatment of hepatic encephalopathy with rifaximin: double blind, double dummy study versus lactulose. Eur J Clin Res. 1993;4:7–18.

[19] Bajaj JS, Riggio O. Drug therapy: rifaximin. Hepatology. 2010;52:1484–8. 20814894 10.1002/hep.23866

[20] Sharma P, Sharma BC. Management of overt hepatic encephalopathy. J Clin Exp Hepatol. 2015;5:S82–7. 26041964 10.1016/j.jceh.2014.04.004 PMC4442855

[21] Hudson M, Schuchmann M. Long-term management of hepatic encephalopathy with lactulose and/or rifaximin: a review of the evidence. Eur J Gastroenterol Hepatol. 2019;31:434–50. 30444745 10.1097/MEG.0000000000001311 PMC6416096

[22] Pratap Mouli V, Benjamin J, Bhushan Singh M, Mani K, Garg SK, Saraya A, et al. Effect of probiotic VSL# 3 in the treatment of minimal hepatic encephalopathy: a non-inferiority randomized controlled trial. Hepatol Res. 2015;45:880–9. 25266207 10.1111/hepr.12429

[23] Ziada DH, Soliman HH, El Yamany SA, Hamisa MF, Hasan AM. Can *Lactobacillus acidophilus* improve minimal hepatic encephalopathy? A neurometabolite study using magnetic resonance spectroscopy. Arab J Gastroenterol. 2013;14:116–22. 24206740 10.1016/j.ajg.2013.08.002

[24] Mittal VV, Sharma BC, Sharma P. Sarin SKJEjog, hepatology: a randomized controlled trial comparing lactulose, probiotics, and L-ornithine L-aspartate in treatment of minimal hepatic encephalopathy. Eur J Gastroenterol Hepatol. 2011;23:725–32. 21646910 10.1097/MEG.0b013e32834696f5

[25] Sharma BC, Singh J. Probiotics in management of hepatic encephalopathy. Metab Brain Dis. 2016;31:1295–301. 27121846 10.1007/s11011-016-9826-x

[26] Dalal R, McGee RG, Riordan SM, Webster AC. Probiotics for people with hepatic encephalopathy. Cochrane database of systematic reviews. 2017. 10.1002/14651858.CD008716.pub3 PMC6464663 28230908

[27] Vilstrup H, Amodio P, Bajaj J, Cordoba J, Ferenci P, Mullen KD, et al. Hepatic encephalopathy in chronic liver disease: 2014 practice guideline by the American Association for the Study of Liver Diseases and the European Association for the Study of the Liver. Hepatology. 2014;60:715–35. 25042402 10.1002/hep.27210

[28] Atif M, Naseem M, Sarwar S, Mukhtar S, Malik I, Hassan MRu, et al. Spectrum of microorganisms, antibiotic resistance pattern, and treatment outcomes among patients with empyema thoracis: a descriptive cross-sectional study from the Bahawal Victoria Hospital Bahawalpur, Punjab, Pakistan. Front Med. 2021;8:665963. 10.3389/fmed.2021.665963 PMC8377472 34422850

[29] Shrestha B, Dunn L. The declaration of Helsinki on medical research involving human subjects: a review of seventh revision. 2019. 10.33314/jnhrc.v17i4.1042 32001865

[30] Xu XY, Ding HG, Li WG, Jia JD, Wei L, Duan ZP, et al. Chinese guidelines on management of hepatic encephalopathy in cirrhosis. World J Gastroenterol. 2019;25:5403–22. 31576089 10.3748/wjg.v25.i36.5403 PMC6767982

[31] Maharshi S, Sharma BC. Prophylaxis of hepatic encephalopathy: current and future drug targets. Hepatol Int. 2024;18:1096–109. 38492132 10.1007/s12072-024-10647-9

[32] Why do we use Lactulose and Rifaximin for Hepatic Encephalopathy? https://www.aasld.org/liver-fellow-network/core-series/why-series/why-do-we-use-lactulose-and-rifaximin-hepatic.

[33] Patidar KR, Bajaj J. Antibiotics for the treatment of hepatic encephalopathy. Metab Brain Dis. 2013;28:307–12. 23389621 10.1007/s11011-013-9383-5 PMC3654040

[34] Saji S, Kumar S, Thomas V. A randomized double blind placebo controlled trial of probiotics in minimal hepatic encephalopathy. Trop Gastroenterol. 2011;32:128–32. 21922877

[35] Muhammad D, Javed M, Ahmed I, Saeed A. To determine the outcome of probiotics in patients of minimal hepatic encephalopathy with liver cirrhosis. Ann Punjab Med Coll. 2017;11:34–7.

[36] Sidhu SS, Sharma BC, Goyal O, Kishore H, Kaur N. L-ornithine L-aspartate in bouts of overt hepatic encephalopathy. Hepatology. 2018;67:700–10. 28749571 10.1002/hep.29410

[37] Sharma BC, Sharma P, Lunia MK, Srivastava S, Goyal R, Sarin SK. A randomized, double-blind, controlled trial comparing rifaximin plus lactulose with lactulose alone in treatment of overt hepatic encephalopathy. Am J Gastroenterol AJG. 2013;108:1458–63. 23877348 10.1038/ajg.2013.219

[38] Rehman HA, Nazar T, Aziz B, Farooq H, Nazar N. Role of probiotics in secondary prophylaxis of hepatic encephalopathy. Pak J Med Health Sci. 2023;17:18–18.

[39] Sakpal TV. Sample size estimation in clinical trial. Perspect Clin Res. 2010;1:67. 21829786 PMC3148614

[40] Chan AW, Tetzlaff JM, Gøtzsche PC, Altman DG, Mann H, Berlin JA, Dickersin K, Hróbjartsson A, Schulz KF, Parulekar WRJB. SPIRIT 2013 explanation and elaboration: guidance for protocols of clinical trials. 2013:346. 10.1136/bmj.e7586 PMC3541470 23303884

