# Supplementary material for: Comparison of efficacy of rifaximin, probiotics and l-ornithine l-aspartate in overt hepatic encephalopathy: a randomized, phase IV, lactulose controlled clinical trial
Source: Trials. 2025 Nov 21;26:534. doi: 10.1186/s13063-025-09173-2 (PMC12639704; doi:10.1186/s13063-025-09173-2)
Supplement: Supplementary file 1 — Additional file 1. Consent Procedure Overview. Additional file 2. Brief Information Sheet. Additional file 3. Study Information for Participants. Additional file 4. Informed Consent Form. [file 13063_2025_9173_MOESM1_ESM.docx]

**CONSENT FORM**

**THE COPE-RPLL TRIAL**

**Title of reserch: Comparison of Efficacy of Rifaximin, Probiotics or LOLA vs. Lactulose alone in Overt Hepatic Encephalopathy: A Phase-IV Trial.**

| Name of PI | Qurratulain Jamil |
| --- | --- |
| Participant name |  |
| Screening ID number |  |

**STATEMENT OF PERSON GIVING CONSENT:**

1. I confirm that I have read the information sheet for the study above, or it has been explained to me in a language I am familiar with.
2. I have spoken with the doctor to my satisfaction, and I've had the chance to ask any questions I had.
3. I understand that my participation is completely voluntary. I have received sufficient information about the research study to decide whether I want to take part.
4. I understand that I have the freedom to withdraw at any time, without giving any explanation and that my medical care or legal rights will not be compromised.
5. I am aware that I will receive a copy of this consent form and the information sheet to keep for my records.
6. I understand that the study staff might look into some of my medical records and I give them permission to do so.
7. I am aware that my data will be available to the public with all personal information omitted.
8. I agree to a copy of this consent form which includes my personal information being made available to Department of Pharmacy Practice, Faculty of Pharmacy, The Islamia University of Bahawalpur for the purpose of monitoring only.
9. I consent to participate in the study mentioned above, the COPE-RPLL trial.

________________________ _________________ ____________________________________

Name of patient Date Signature/ thumbprint or other mark (if unable to sign)

________________________ _________________ ____________________________________

Name of witness Date Signature

(witness if patient is unable to read or write)

**STATEMENT OF PERSON OBTAINING INFORMED CONSENT**

I have provided a detailed explanation of this research to the participant, including the risks and benefits to make an informed decision.

________________________ _________________ ____________________________________

Name Date Signature

Chictr.or.cn ID: ChiCTR2300075925

Informed Consent Form Version 1.0 Page 1 of 1

**Consent procedure overview**

Patients age > 18 years old, admitted in hospital with diagnosis of OHE. No contraindication for any trial drugs.

YES

NO

Either patient is willing to be considered for inclusion in the trial or if he/she is incapable to respond, assent will be collected from first degree relative of legally authorized representative.

If not eligible, not wish to participate, or in case of he/she is incapable to respond, his/her first degree relative or legally authorized representative are not giving assent please refrain from including yourself.

NO

YES

- Complete information is provided to the patient or first degree relative/LAR and researcher will obtain written informed consent/assent.
- Baseline data of patient collected
- Eligibility confirmed
- Patients randomized and treatment will be administered.

]

**If any patient/LAR/First degree relative is unable to read or write:**

- Provide trial information to the patient/LAR/First degree relative in the presence of an independent witness
- A mark, such as a thumbprint, will be collected from the patient/LAR/First degree relative on the trial consent/assent form
- The independent witness is required to sign a consent/assent form.

**In all cases**

- The researcher who is acquiring consent/assent is required to sign the consent/assent form.
- Place the original consent/assent form in the Investigator’s Site File.
- Provide a copy of the signed consent/assent form to the patient /LAR/First degree relative
- The consent process should be recorded in the patient medical notes.

**THE COPE-RPLL TRIAL**

This hospital is participating in a research study aimed at finding which treatment group is most efficacious in treating overt hepatic encephalopathy in patient of DCLD. This leaflet outlines the reasons for conducting this study and details what it entails.

**What is the COPE-RPLL trial?**

The COPE-RPLL trial is a study designed to evaluate whether lactulose alone is better than interventional adjunct therapy to combat overt hepatic encephalopathy. It is a four arms study containing group A (lactulose) as controlled group and adjunct therapies including group B (rifaximin + lactulose), group C (probiotics + lactulose) and group D (LOLA (l-ornithine l-aspartate + lactulose).

**What is overt hepatic encephalopathy?**

Overt hepatic encephalopathy (OHE) is a serious type of hepatic encephalopathy (HE), which refers to brain dysfunction resulting from acute or chronic liver disease. It manifests through various neurological and psychiatric symptoms, such as confusion, disorientation, sleep pattern changes, lethargy, unusual movements, changes in personality, extreme anxiety and may result into coma.

**What are our trial interventional drugs?**

1. **Lactulose:**

Lactulose is synthetic, non-absorbable sugar that works like a liquid laxative. Its mechanism of actions includes breakdown of lactulose in the colon by gut bacteria into acetic and lactic acid which causes the gut to become more acidic which intern promotes the conversion of NH3 into non-absorbable NH4+ and as a result, NH4+ gets trapped in the colon helping to lower ammonia levels in the blood.

1. **Rifaximin:**

Rifaximin is a non-absorbable oral antibiotic that is selective to gut. It reduces the population of ammonia-producing bacteria in the gut, thereby decreasing the amount of ammonia absorbed into the bloodstream.

1. **Probiotics:**

Probiotics are live bacteria and when they are consumed, they contribute to treatment of HE by modifying the gut microbiota, reducing the presence of harmful bacteria, acidifying the intestinal mucosa, reducing the production and absorption of ammonia and lowering endotoxin levels.

1. **L-Ornithine-L-aspartate (LOLA)**

LOLA is a mixture of two amino acids, l-ornithine and l-aspartate. It can be taken orally or as injectable. It can lower blood ammonia levels by promoting both the urea cycle and glutamine synthesis in periportal hepatocytes. Intravenous LOLA can be employed as an add on therapy for patients who do not respond to standard therapy.

**Why are we doing this trial?**

Lactulose is given as alone therapy in treating OHE in most LMIC. However, different treatment options are available for combating OHE according to the international guidelines. Hence, we want to compare which therapy is efficacious, lactulose alone or other treatment options in combination with lactulose.

There are number of studies carried out comparing one treatment to other but there is no study comparing all four treatment groups together i.e. group A (lactulose) as controlled group, group B (rifaximin + lactulose), group C (probiotics + lactulose) and group D (LOLA (L-Ornithine L-Aspartate) + lactulose). This study would be the first randomized controlled trial (RCT) to fill this gap of research according to our best knowledge.

**What does the trial involve?**

Joining our trial won’t influence your plans for usual care for treatment. You will continue to receive the regular care offered to every patient of OHE. There are no limitations on the use of additional medications with trial medications. Trial participation will not lead to any necessary treatments being prohibited. Additionally, taking part in the study is completely free, you will not have to pay anything.

We will examine your medical records and might ask you some questions to confirm if you have symptoms of OHE. Consent for participation may be obtained from you. If you are not able to respond, your first degree relative or LAR may assent for your participation.

After obtaining consent or assent, you will be randomly allocated to receive the trial treatment groups according to your enrolment schedule. Following that, you will get the required treatment either orally or by NG tube or in vein. After 5 days of participation in the trial, improvement in mental state grade will be observed by West Haven Criteria. We will also observe length of hospital stay and time of recovery in days from participation in the trial.

Trial drugs have been used for years without showing any harmful effects. Some minor effects include flatulence, diarrhea and abdominal discomfort.

This study is organized by Department of Pharmacy Practice, Faculty of Pharmacy, The Islamia University of Bahawalpur, Pakistan and is supervised by QurratulAin Jamil, contact information is given below:

Name:

Address:

Phone:

Email:

[Hospital Contact Details]

Name of PI

Name of Hospital

Hospital Address

Contact Phone Number

**The COPE-RPLL trial**

Comparison of Efficacy of Rifaximin, Probiotics or LOLA vs. Lactulose alone in Overt Hepatic Encephalopathy: A Phase IV Trial.

**STUDY INFORMATION FOR PARTICIPANTS**

| **We invite you to take part in a research study called COPE-RPLL** | **Contents** |
| --- | --- |
| - Before your decision to take part, we would like to clarify the reasons for conducting the study and what it will involve. | 1. What is this study for? 2. Why are you asking me to take part? 3. What will happen if I take part? |
| - Kindly read the information or ask any queries from attending physicians and trial pharmacist. You can ask any query you like to ask before enrolling yourself in the trial. | 1. How long will I be in the study? 2. Will I benefit from taking part?   6. Could I be harmed by taking part?  7. Can I change my mind about taking part?  8. What happens afterwards? |
| - Participating is entirely up to you. If you decide not to join, the attending physicians will still provide you with all the usual care offered at this hospital. | 9. What information do we keep private?  10. Who is doing this study?  11. Who has reviewed the study?  12. Who can I contact about any questions?  13. What else do I need to know? |

1. **What is the study for?**

In Pakistan the rate of HCV and HBV is high which ultimately causes cirrhosis leading to a condition known as hepatic encephalopathy in approximately 30%–45% of cirrhotic patients. We are doing study on overt hepatic encephalopathy in which patient presents flapping tremors and mental alterations leading to coma which disrupts the daily activities and affects patient health-related quality of life (HRQOL). Additionally, it increases the risk of car accidents for those affected due to their reduced attention and slower reaction times.

In this study, we aim to compare which trial group is most efficacious in grade reversal of overt hepatic encephalopathy (OHE): group A (lactulose) as controlled group, group B (rifaximin + lactulose), group C (probiotics + lactulose) and group D (LOLA (L-Ornithine L-Aspartate) + lactulose).

Lactulose is first-line of treatment for patients of OHE and now it is used in adjunct with other treatment options including rifaximin, LOLA and probiotics. This prompted us to consider comparison among all 4 treatment groups together mentioned above. All medicines we are using have completed phase 4 trial and the risk of adverse effects is also minor. All the trial medicines are recommended in guidelines AASLD and Pakistan Society of Hepatology Guidelines.

In spite of availability of several treatment options for combating OHE, according to the international guidelines, it is not conclusive that which treatment group is better in treating OHE. We hope that this study will find which treatment group is better in treating OHE of different grades.

1. **Why are you asking me to take part?**

We are inviting you to participate because your physician believes you are diagnosed with OHE. To be eligible, you must be at least 18 years old. The choice to participate is entirely up to you.

1. **What will happen if I take part?**

Participating in the study will not affect your treatment plan. You will receive all the usual care provided to every patient of OHE at your hospital. The treatment related to the study is free and there will be no costs for you to participate.

If you decide to participate in study, we will request that you complete a consent form. After that, we will gather information about you and any of your old treatment plan. You will be randomly allocated any of the study treatment group and study medicines will be administered either by mouth, NG tube or IV route. We will monitor reversal in your OHE grades for consecutive 5 days and will also ensure about safety of study medicines.

1. **How long will I be in the study?**

You will be in study for 5 days, till date of discharge or till 28 days from date of participation in clinical trial whichever occur earlier.

1. **Will I benefit from taking part?**

We cannot determine whether participating in this study will provide any personal benefit to you or not. We hope that this study will find which treatment group is better in treating OHE of different grades. If you take part, you may get adjunct therapy in addition to lactulose (usual therapy) randomly or you may get lactulose only if you are in control group. If you don’t take part, you will get the usual treatment with lactulose which is in practice in hospital.

By participating, we aim for you to gain more knowledge for the future, which you can then share with others or you can tell how you feel by taking adjunct therapy for your condition. In the future, the findings from this study will aid doctors in improving care for OHE patients.

1. **Could I be harmed by taking part?**

Many guidelines (AASLD, Pakistan Society of Hepatology Guidelines) recommend our trial medicines for treating OHE and these are widely used for years. A lot of studies prove no serious side effects with any of our trial medications. Only minor include flatulence, diarrhea and abdominal discomfort. Your attending physician will monitor your vitals, providing the highest level of care if any issue arises. They will also inform the study team about any problem that occur.

1. **Can I change my mind about taking part?**

Yes, you can withdraw from the study whenever you want. Just let us know by saying something like, “I don’t want to be in this study anymore.” Your attending physician and hospital staff will continue to provide you usual care. If you have any health problems after you decide to leave the study, please let us know about these.

1. **What happens afterwards?**

We will keep your results in our custody. If you want to receive the results of this study, please inform the study doctor and they will ensure you get a copy.

1. **What information do we keep private?**

We will maintain the privacy of all information gathered about you and will store it securely. Only the study staff will have access to this information at Department of Pharmacy Practice, Faculty of Pharmacy, The Islamia University of Bahawalpur, Pakistan. All information related to your initials (consent form) will be destroyed or your personal information will be removed right after their use.

We will publish the study results so that physicians and researchers can benefit from the findings. Your personal information will not be included in any reports, ensuring your anonymity. The study team may share the data with other researchers and the public, but all personal details will be removed before that.

1. **Who is doing this study?**

A group of doctors, nurses and researchers are working together from Bahawal Victoria Hospital and Department of Pharmacy Practice, Faculty of Pharmacy, The Islamia University of Bahawalpur, Pakistan for finding the best treatment options for combating overt hepatic encephalopathy.

1. **Who has reviewed the study?**

Pharmacy Human Ethics Committee (PHEC) of the Islamia University of Bahawalpur has thoroughly reviewed and approved to conduct this study.

Name: Islamia University of Bahawalpur

Address: Islamia University of Bahawalpur, Khawaja Fareed campus, Pakistan.

Website: Iub.edu.pk

1. **Who can I contact about any questions?**

If you have any questions regarding the study, feel free to ask to speak with the study team and they will do their best to help you.

1. **What else do I need to know?**

Neither Bahawal Victoria Hospital nor Department of Pharmacy Practice, Faculty of Pharmacy, The Islamia University of Bahawalpur, Pakistan, produces trial medicines including (Rifaximin, LOLA, Probiotics and Lactulose). Signing the consent form will not impact your legal rights to pursue compensation.
